# Investigating Urinary Circular RNA Biomarkers for Improved Detection of Renal Cell Carcinoma

**DOI:** 10.3389/fonc.2021.814228

**Published:** 2022-01-31

**Authors:** Madonna R. Peter, Fang Zhao, Renu Jeyapala, Shivani Kamdar, Wei Xu, Cynthia Hawkins, Andrew J. Evans, Neil E. Fleshner, Antonio Finelli, Bharati Bapat

**Affiliations:** ^1^ Lunenfeld-Tanenbaum Research Institute, Sinai Health System, Toronto, ON, Canada; ^2^ Department of Laboratory Medicine & Pathobiology, University of Toronto, Toronto, ON, Canada; ^3^ Institute of Medical Science, University of Toronto, Toronto, ON, Canada; ^4^ Department of Biostatistics, Princess Margaret Cancer Centre, University Health Network, Toronto, ON, Canada; ^5^ Arthur and Sonia Labatt Brain Tumor Research Centre, Hospital for Sick Children, Toronto, ON, Canada; ^6^ Department of Laboratory Medicine, Mackenzie Health, Richmond Hill, ON, Canada; ^7^ Division of Urology, Department of Surgery, Princess Margaret Cancer Centre, University Health Network, Toronto, ON, Canada

**Keywords:** renal cell carcinoma, ccRCC, circular RNA, biomarkers, liquid biopsy

## Abstract

Renal cell carcinomas (RCC) are usually asymptomatic until late stages, posing several challenges for early detection of malignant disease. Non-invasive liquid biopsy biomarkers are emerging as an important diagnostic tool which could aid with routine screening of RCCs. Circular RNAs (circRNAs) are novel non-coding RNAs that play diverse roles in carcinogenesis. They are promising biomarkers due to their stability and ease of detection in small quantities from non-invasive sources such as urine. In this study, we analyzed the expression of various circRNAs that were previously identified in RCC tumors (circEGLN3, circABCB10, circSOD2 and circACAD11) in urinary sediment samples from non-neoplastic controls, patients with benign renal tumors, and clear cell RCC (ccRCC) patients. We observed significantly reduced levels of circEGLN3 and circSOD2 in urine from ccRCC patients compared to healthy controls. We also assessed the linear variant of EGLN3 and found differential expression between patients with benign tumors compared to ccRCC patients. These findings highlight the potential of circRNA markers as non-invasive diagnostic tools to detect malignant RCC.

## Introduction

Renal cell carcinoma (RCC) is the most common malignancy of the kidney, accounting for approximately 2% of global cancer diagnoses and deaths in 2020 (1, 2). Survival with RCC is dependent on clinical stage at first diagnosis, with 5-year survival rate around 90% for localized disease and 12% for metastatic disease (3). RCC tumor subtypes range from less aggressive chromophobe, papillary type I (pRCC type I) to more aggressive pRCC Type II and clear cell RCC (ccRCC) (4). Among these, ccRCC constitute the major tumor subtype (~70%) with high metastatic potential (5).

Early detection of ccRCC is challenging as more than 50% of RCC tumors are asymptomatic and frequently detected as an incidental finding during abdominal imaging for other conditions (6). Approximately 30% of ccRCC patients are first diagnosed with metastatic disease with poor overall prognosis and survival (7). RCC is often treated with surgery based on imaging and/or needle biopsy diagnosis; however, current imaging techniques cannot accurately predict tumor histopathology (6). Furthermore, tissue-based immunohistochemical markers are insufficient for differential diagnosis (8). Several tissue-based miRNA and protein markers have been identified, but none have impacted patient management to date (9). Identifying molecular markers in liquid biopsies (i.e. serum, urine) is a promising approach, which can potentially circumvent issues of tumor tissue heterogeneity and overcome limitations in tissue availability (10). In this regard, non-invasive biomarkers that could be routinely used to screen for RCC at an early stage would be important for the clinical management of RCC.

Circular RNAs (circRNAs) are a class of endogenous noncoding RNAs associated with wide-ranging functions including regulation of key cancer driver genes (11). They are distinct from linear RNA and are produced by covalent linking of 5’ and 3’ ends of an RNA molecule (12). CircRNAs are abundant, stable and associated with tissue-specific and cell-specific expression patterns (13). Differential expression of these circRNAs has been demonstrated in multiple cancer types and can be detected in a variety of sources, including tumor tissue and biofluids (14–17). Several circRNAs have already been identified as differentially expressed in ccRCC tumors compared to matched benign tissue (18–21). These altered circRNAs could potentially serve as diagnostic markers to facilitate early detection of malignant disease. In this study, we selected circRNAs that were previously shown to be upregulated in ccRCC tumors and assessed whether these markers could be detected in urine samples from patients with confirmed diagnosis of ccRCC.

## Materials and Methods

### Patient Cohort and Urine Sample Collection

All patients were previously recruited by the University Health Network (UHN) McCain genitourinary biobank (MGB). Informed written consent was obtained in accordance with approved research ethics board protocols by UHN and Sinai Health System (SHS). Urine samples were collected from 19 healthy non-neoplastic/normal patients, 8 patients with benign renal tumors and 76 patients with confirmed diagnosis of ccRCC. For ccRCC patients, all samples were collected prior to any surgical intervention. Each patient provided approximately 20-90mL of urine, which was then spun at 400 x *g* for 10 minutes at 4°C. The urinary sediment was stored with 2mL of supernatant in cryovials and snap frozen in liquid nitrogen. Patients’ clinical data at diagnosis (sex, clinical and pathological stage, Fuhrman grade) and follow-up metastasis status were also collected (summarized in **Table 1**).

**Table 1:** Summary of cohort clinical characteristics.

Clinical characteristic	Normal	Benign	ccRCC
Sex			
Male	9	2	53
Female	10	6	23
Fuhrman Grade			
G1	n/a	n/a	10
G2	n/a	n/a	40
G3	n/a	n/a	23
G4	n/a	n/a	3
Clinical Stage (TNM) at diagnosis			
T1	n/a	n/a	51
T2	n/a	n/a	3
T3	n/a	n/a	18
T4	n/a	n/a	3
Unknown T	n/a	n/a	1
Nx	n/a	n/a	65
N0	n/a	n/a	9
N1	n/a	n/a	1
Unknown N	n/a	n/a	1
Mx	n/a	n/a	20
M0	n/a	n/a	51
M1	n/a	n/a	5
Metastasis status post-diagnosis			
Yes	n/a	n/a	16
No	n/a	n/a	60

n/a, not applicable.

### Purification of Total RNA and cDNA Conversion

Frozen urinary sediment samples were thawed, and total RNA was extracted using the QIAamp Circulating Nucleic Acid Kit (Qiagen, Hilden, Germany), specifically using the protocol for urine miRNA extraction. Contaminating DNA was digested using the Qiagen RNase-Free DNase Set followed by clean-up with the RNeasy mini kit according to manufacturer’s protocol. All samples were eluted with 15μL of elution buffer and reverse transcribed using the Superscript VILO cDNA synthesis kit (ThermoFisher Scientific, Waltham, MA, USA).

### qPCR Assays and Data Analysis

Using previously published datasets, we selected circRNA candidates and a linear RNA candidate that were shown to be upregulated in ccRCC tumor tissue compared to matched benign tissue (18–21). These included: circEGLN3, linear EGLN3, circABCB10, circSOD2, and circACAD11. We also selected circHIPK3, which is dysregulated in multiple cancer types, including colorectal cancer and gastric cancer (22–24). We used GAPDH mRNA as an internal control to normalize expression for each candidate RNA marker (25). Primer sequences for circRNA or linear RNA targets were either previously published or designed specifically for this study (**Supplementary Table 1**) and qPCR assays were optimized with RNA from HEK 293 cell lines. For circRNA candidates, all primers were divergent, with some specifically located on the circRNA junctional sites.

For qPCR assays, 1μL of converted cDNA sample was used for each assay, with two replicates per sample. The Power SYBR green master mix (ThermoFisher Scientific) was used, with 300nM of primers per reaction. All qPCR assays were performed on the ThermoFisher Scientific QuantStudio6Flex real-time PCR system (95°C for 10 min, 40 cycles of 95°C for 15 sec and 60°C for 1 min, followed by melt-curve analysis). GAPDH was used as a normalizing control, with HEK 293 derived cDNA as the positive control. Non-template controls were utilized for all assays. Detection of circRNA junctional sequences were confirmed by Sanger sequencing (service provided by The Centre for Applied Genomics, Hospital for Sick Children).

The delta Ct for each candidate circRNA/linear RNA was calculated using GAPDH to assess relative expression between normal, benign and ccRCC samples. If there was no amplification of a candidate marker in a sample, the marker was considered as non-detectable and Ct value was set to the maximum (Ct 40). The Kruskal-Wallis test was used to compare delta Ct values between the three groups, followed by pairwise comparisons with Benjamini-Hochberg FDR correction. For each candidate RNA marker, the delta-delta Ct method was next used to assess differences between benign and ccRCC patients, with normal patients serving as the control group. Significantly altered markers between benign and ccRCC patients were identified by the Mann-Whitney test. Receiver operating characteristic (ROC) curve analysis was performed using the ROCR package. All statistical tests were two-sided and conducted using R v4.1.1.

## Results

### Optimization of Candidate circRNA Detection Assays in Urinary Sediment Samples

We first assessed whether the candidate markers could be detected in HEK 293 cells and urine samples from 8 ccRCC patients. We confirmed detection of the unique junction sites of all circRNA candidates using Sanger sequencing (**Supplementary Figure 1**). Additional variants of circEGLN3 and circABCB10, as well as another candidate circRNA (circAGAP1) were also tested but not detectable in urinary sediment samples (20, 21, 26). Interestingly, two circRNA candidates, circABCB10 and circACAD11, were detectable in genomic DNA (gDNA) from HEK 293 cell lines. We assessed urine-derived gDNA samples from RCC patients and saw similar melt curve profiles for circABCB10 and circACAD11 as the equivalent RNA/cDNA samples (**Supplementary Figure 2**). The circRNA junction sequences for circABCB10 and circACAD11 were also confirmed in gDNA samples by Sanger sequencing. To eliminate the possibility of RNA contamination, gDNA samples were treated with RNase A, which would digest linear RNA and circRNA. However, these junctional sequences were still detectable in DNA samples post RNase A treatment (data not shown). Although these results are interesting and potentially indicative of circular DNA, we performed DNase digestion of all RNA samples from patients to mitigate any potential DNA contamination.

### Assessment of circRNA Expression in Urinary Sediments From Normal, Benign and ccRCC Patients

We next performed qPCR analysis of all urinary sediment samples from 19 healthy control/normal patients, 8 patients with benign tumors, and 76 patients with ccRCC. While certain candidates (linear EGLN3, circHIPK3, and circSOD2) demonstrated detectable levels in all urinary sediment samples analyzed, circABCB10 was detected in 97% of samples, circEGLN3 in 69% of samples, and circACAD11 in 60% of samples.

**Figure 1:**
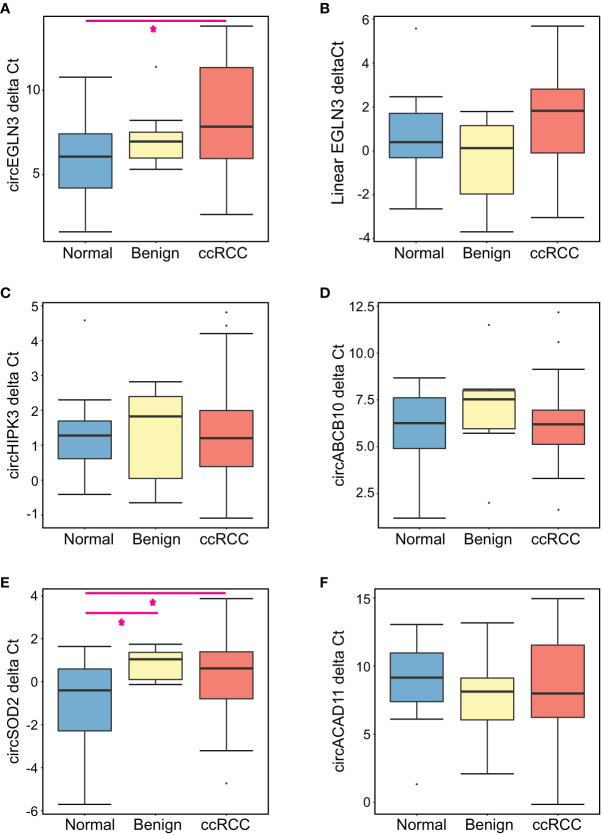
Summary of expression levels of all candidate markers for each patient group. The delta Ct for each candidate RNA marker was calculated using GAPDH Ct values for all urine samples analyzed. Boxplots show the median delta Cts as well as the first and third quartile for **(A)** circEGLN3, **(B)** linear EGLN3, **(C)** circHIPK3, **(D)** circABCB10, **(E)** circSOD2, and **(F)** circACAD11. Kruskal-Wallis analysis was performed to compare all patient groups, with pairwise comparisons for significantly altered RNA markers (*p < 0.05).

The expression levels of each candidate marker relative to GAPDH (delta Ct) is summarized in **Figure 1**. When comparing all three patient groups, circEGLN3 levels were significantly different between the normal (non-neoplastic) group versus ccRCC patients (Kruskal-Wallis test, p < 0.05) (**Figure 1A**). Interestingly, the median delta Ct value of circEGLN3 (normalized by GAPDH) was higher in ccRCC patient urine samples compared to normal samples. That is, there were lower detectable levels of circEGLN3 in ccRCC urine samples compared to normal controls. This was a surprising finding since increased expression of circEGLN3 has been observed in tumor tissue from ccRCC patients compared to matched benign tissue (18, 21). Similarly, there were lower levels of circSOD2 in urine samples from benign and ccRCC patients compared to healthy controls (**Figure 1E**).

**Figure 2:**
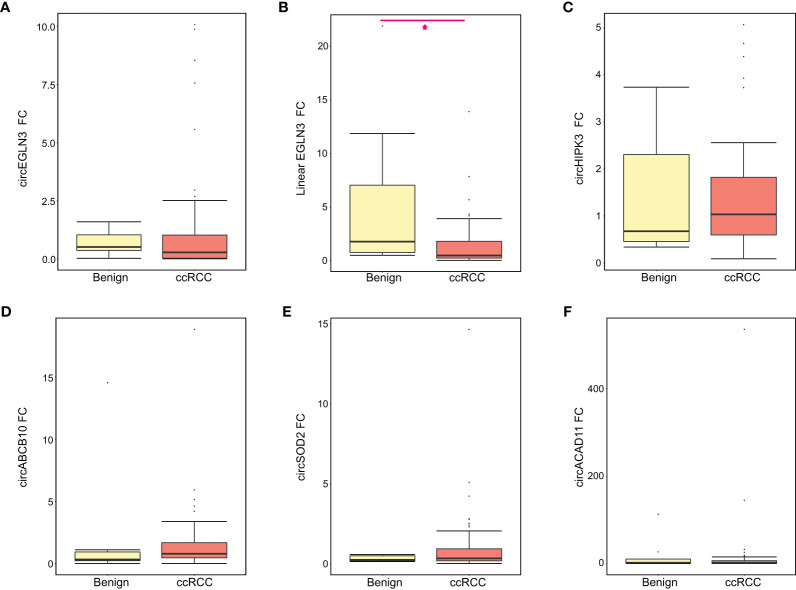
Relative expression of all RNA candidates between benign and ccRCC groups. The relative fold-change (FC) for each RNA candidate was calculated for benign and ccRCC samples using normal samples as the control group. Boxplots show the median FC, first and third quartile for **(A)** circEGLN3, **(B)** linear EGLN3, **(C)** circHIPK3, **(D)** circABCB10, **(E) **circSOD2, and **(F)** circACAD11. Mann-Whitney test results are shown (*p < 0.05).

Using normal samples as a control group, we calculated the relative fold-change for each candidate and compared expression levels between the benign and ccRCC groups (**Figure 2**). We observed increased linear EGLN3 expression in urine from patients with benign tumors (relative to healthy controls) compared to ccRCC patients (**Figure 2B**). In addition, we performed ROC analysis to assess the diagnostic potential of these candidate markers. We found that detected circEGLN3 and circSOD2 levels had an AUC of 0.71 and 0.68, respectively, for separating cancer patients versus non-neoplastic patients (**Supplementary Figure 3**). These markers would need to be validated in additional cohorts to confirm their utility as diagnostic tools.

We further examined expression levels among ccRCC patients using additional clinical parameters, including Fuhrman grade, TNM staging at diagnosis, and current metastasis status. For Fuhrman grade, there was no significant difference in detection levels of any candidate circRNAs (**Supplementary Figure 4**). Similar findings were observed when analyzing clinical T stage at first diagnosis of ccRCC (**Supplementary Figure 5**). We also obtained metastasis status at first diagnosis of ccRCC for 56 patients (**Table 1**). We found significantly less circACAD11 in urine from patients with metastasis at the time of diagnosis compared to those without metastasis (p<0.05, Mann-Whitney Test) (**Supplementary Figure 6**). We next examined post-diagnosis metastasis status and found no significant differences in circRNA or linear RNA levels (**Supplementary Figure 7**).

## Discussion

Liquid biopsies offer promising minimally invasive strategies to detect malignancies, track molecular alterations associated with disease progression, and potentially predict therapeutic response (27–32). In particular, detection of circulating DNA and RNA markers in blood and urine samples are an area of active study in ccRCC (10, 33, 34). Our study has demonstrated that select RCC-related circRNAs can be detected in urine samples from ccRCC patients, and their differential expression is associated with ccRCC status. We have also demonstrated that these markers can be successfully analyzed in patients’ urine samples thereby providing a non-invasive strategy for future studies. This work highlights the diagnostic potential of circSOD2, circEGLN3 and linear EGLN3 for early detection of ccRCC.

In this study, we focused on urinary samples collected around first diagnosis of ccRCC and prior to surgical intervention. We found decreased levels of circELGN3 and circSOD2 in urine from ccRCC patients compared to healthy controls. These candidates were previously shown to be upregulated in ccRCC tissue compared to matched control tissue, especially circEGLN3, which has demonstrated dysregulated expression in a few studies (18, 21, 26). The reason for these divergent expression profiles between urine and tumor tissue is unclear. Furthermore, the relative abundance of these circRNAs in non-neoplastic tissues from ccRCC patients compared to normal renal tissue from non-cancer patients is not known. Such a comparison may shed light on variations observed in circRNA expression levels in tumor tissue versus urine samples from ccRCC patients. Further separation of exosomes in urine may also help to improve detection of these markers and the ability to distinguish ccRCC from benign tumors (35).

The precise functional role(s) of these circRNAs in the development of malignant disease remain to be characterized. However, recent evidence suggests that silencing of circEGLN3 could reduce RCC cell proliferation and invasion (36). Another interesting observation was the presence of circRNA junctions in DNA from HEK 293 cells and urine samples. This could be an example of extrachromosomal circular DNA (37), but additional studies are required to further explore any connection between circRNA and circDNA. Overall, our exploratory study has found that circRNAs may serve as diagnostic markers and their detection in urine samples offer a non-invasive strategy to identify ccRCC. Additional validation in a larger series of patients is needed to confirm the utility of these circRNAs in the clinical management of aggressive disease.

## Data Availability Statement

The original contributions presented in the study are included in the article/**Supplementary Material**, further inquiries can be directed to the corresponding author.

## Ethics Statement

The studies involving human participants were reviewed and approved by University Health Network and Sinai Health System. The patients/participants provided their written informed consent to participate in this study.

## Author Contributions

BB designed the overall study. WX and CH contributed to statistical and circRNA analysis aspects of study. MP performed qPCR assays for all samples and data analysis. FZ and RJ contributed to the optimization of qPCR assays. FZ and SK assisted with data analysis of the cohort. NF, AE, and AF performed patient recruitment and follow-up. MP and BB composed the manuscript with feedback from all co-authors involved. All authors contributed to the article and approved the submitted version.

## Funding

This study was funded by a Give for a Cure Grant #24205 from the Cancer Research Society and a KCRNC-KCC-CUASF award.

## Conflict of Interest

The authors declare that the research was conducted in the absence of any commercial or financial relationships that could be construed as a potential conflict of interest.

## Publisher’s Note

All claims expressed in this article are solely those of the authors and do not necessarily represent those of their affiliated organizations, or those of the publisher, the editors and the reviewers. Any product that may be evaluated in this article, or claim that may be made by its manufacturer, is not guaranteed or endorsed by the publisher.
