# Supplementary material for: Investigating Urinary Circular RNA Biomarkers for Improved Detection of Renal Cell Carcinoma
Source: Front Oncol. 2022 Jan 31;11:814228. doi: 10.3389/fonc.2021.814228 (PMC8841801; doi:10.3389/fonc.2021.814228)
Supplement: Supplementary file 1 [file DataSheet_1.zip › Supplementary_files/Supplementary_Figures.pdf]

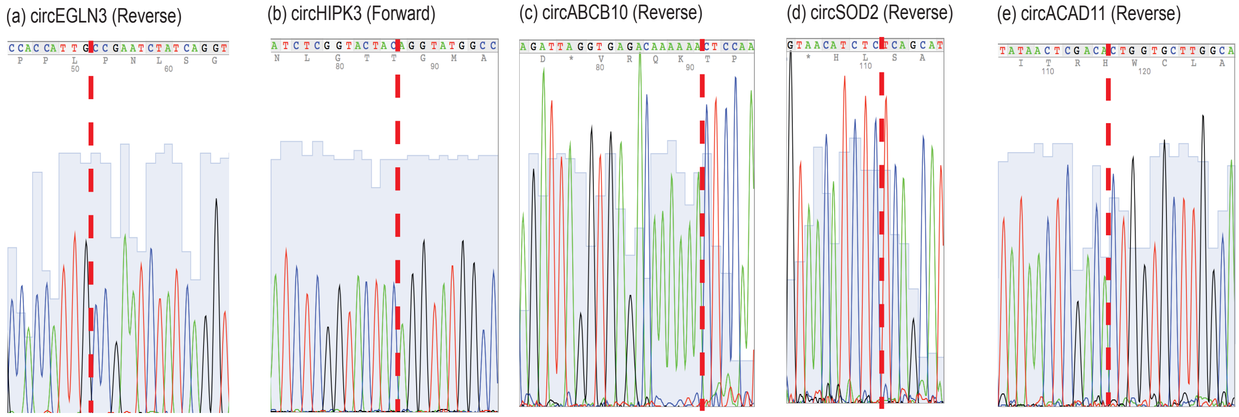

Supplementary Figure 1. Sanger sequencing chromatograms of candidate circRNA junctions.

For (a) circEGLN3, (b) circHIPK3, (c) circABCB10, (d) circSOD2, and (e) circACAD11 the junctional site (dotted red line) sequences were confirmed in HEK 293 cells and urine sediment samples. Example chromatograms are shown for each circRNA with primer used for sequencing indicated.

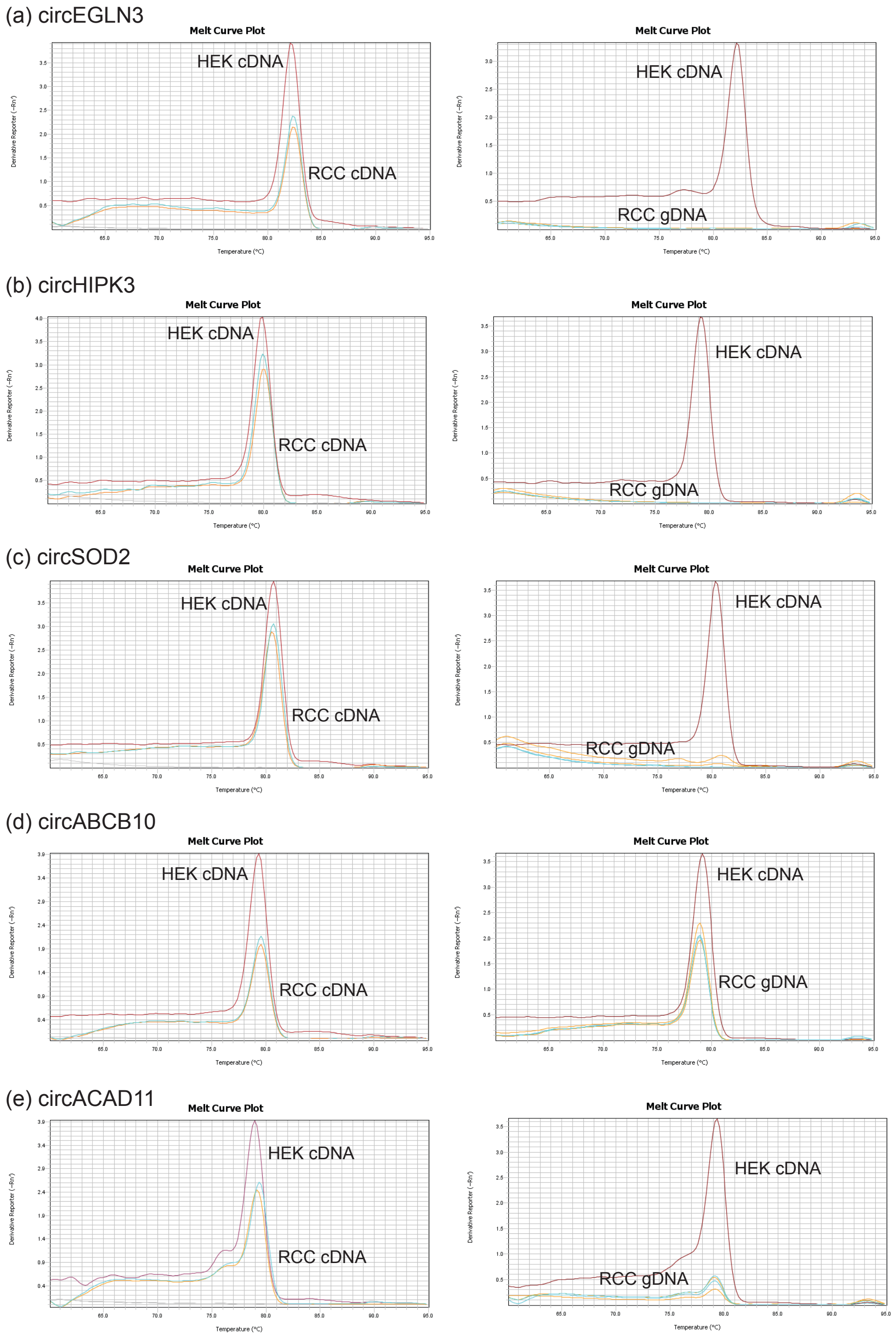

Supplementary Figure 2. Melt-curve analysis comparing cDNA and genomic DNA. Melt curves of cDNA from HEK 293 cells and RCC urine samples were analyzed to ensure detection of the correct amplicon for all samples. Genomic DNA (gDNA) from RCC urine sediments were also tested and compared with expected cDNA melt curves. Grey lines = non-template controls.

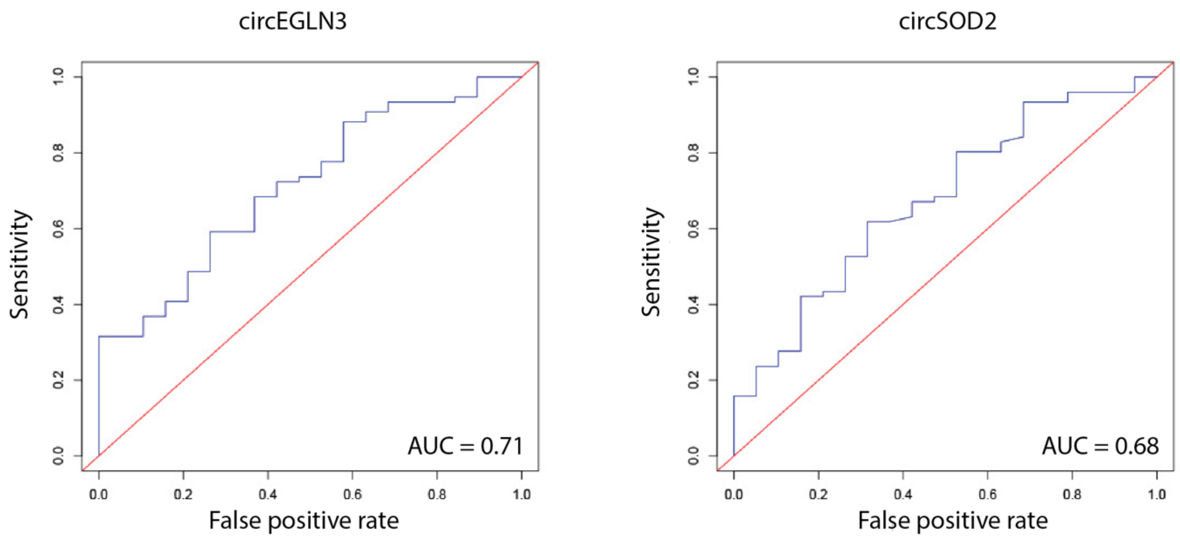

Supplementary Figure 3. ROC analysis of circEGLN3 and circSOD2.
ROC analysis was performed to assess whether circEGLN3 and circSOD2 expression levels could distinguish between ccRCC patients versus normal/non-neoplastic patients. Area under the curve (AUC) values are shown for each marker.

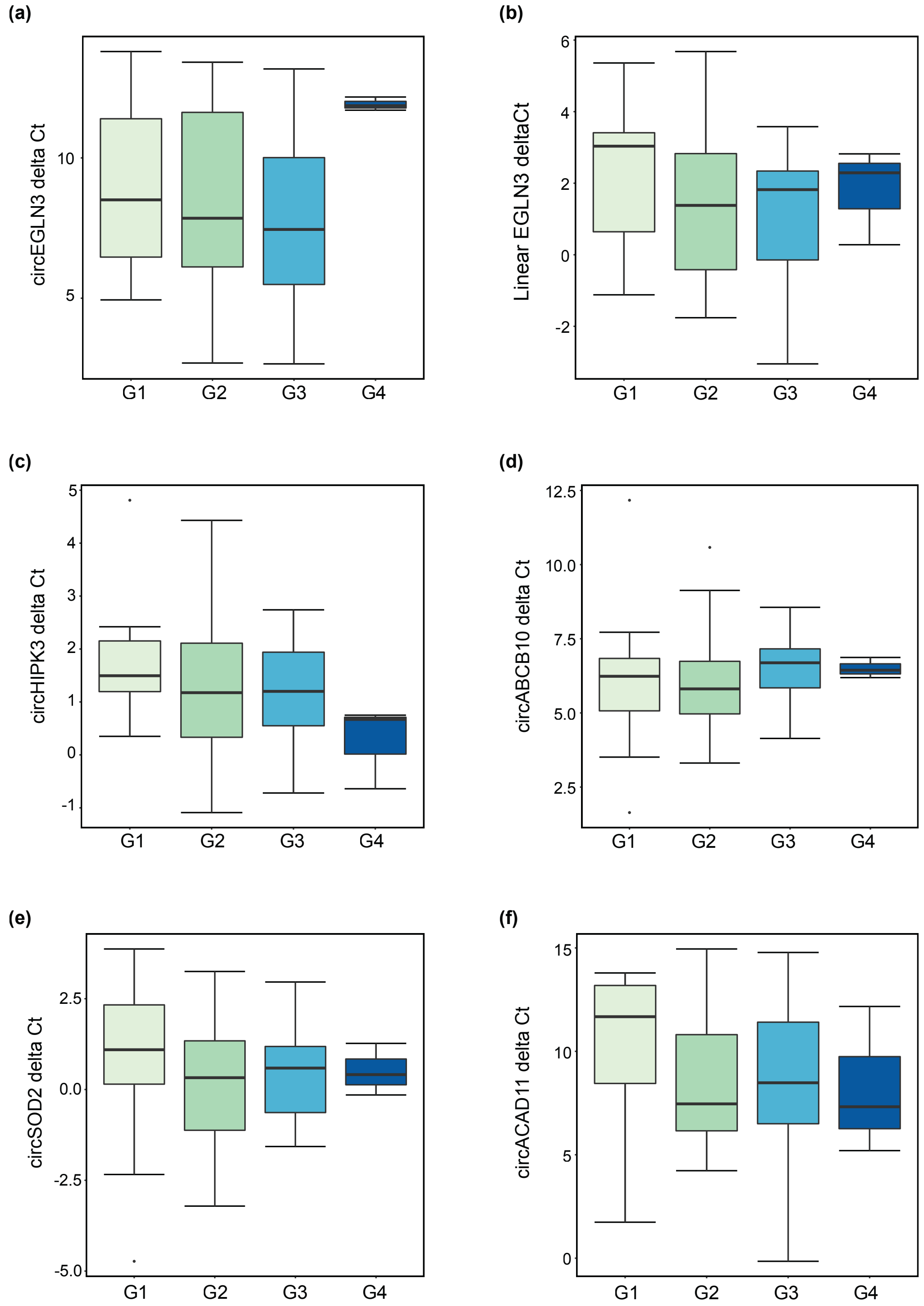

Supplementary Figure 4. Relative expression of all candidates stratified by Fuhrman grade. The delta Ct for each RNA candidate is plotted according to Fuhrman grade in ccRCC patients. Boxplots show the median, first and third quartile values. Kruskal-Wallis analysis was performed to compare all groups.

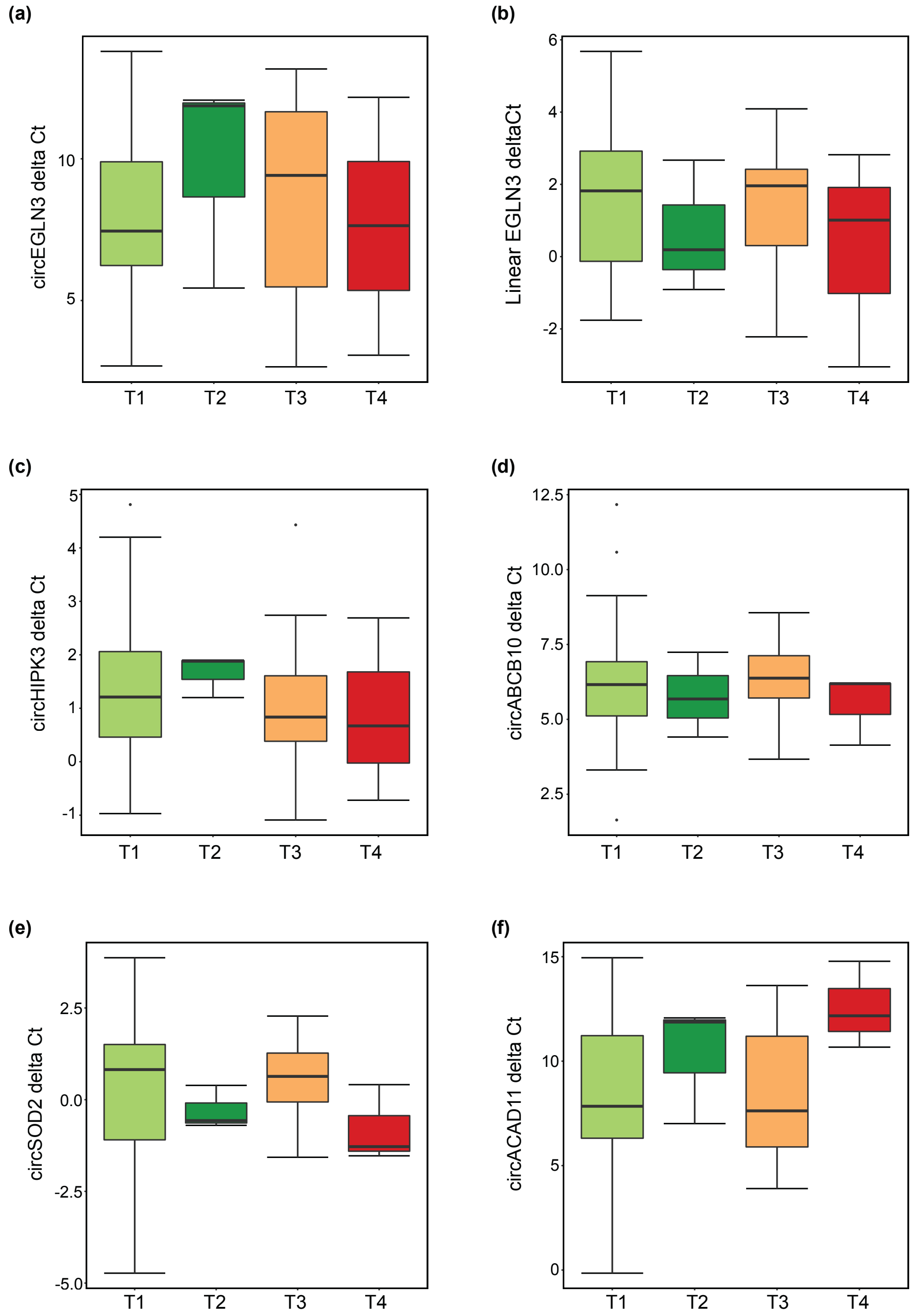

Supplementary Figure 5. Relative expression of all candidates stratified by clinical T stage. The delta Ct for each RNA candidate is plotted according to T stage at diagnosis in ccRCC patients. Boxplots show the median, first and third quartile for all candidates. Kruskal-Wallis analysis was used to compare all groups.

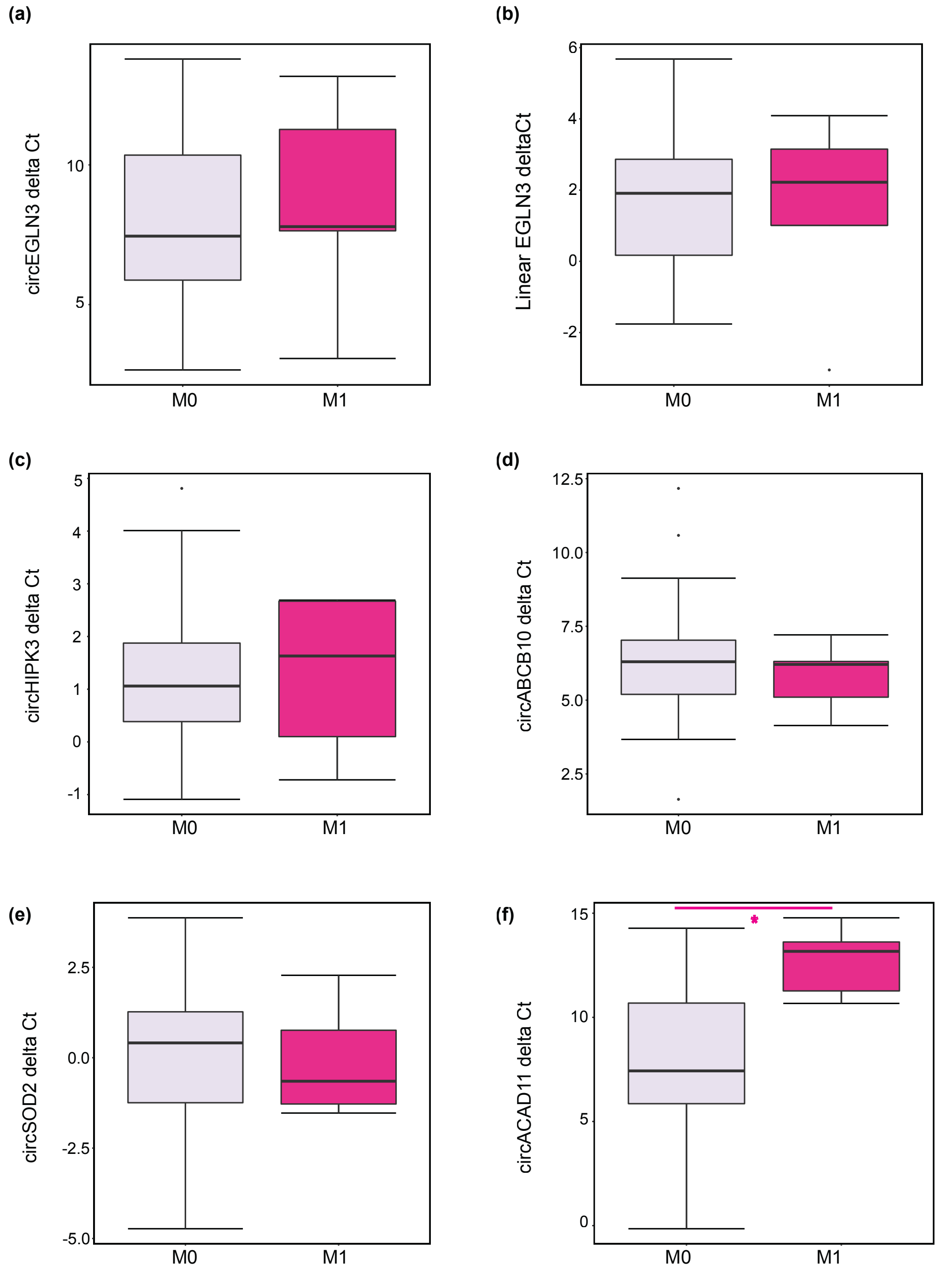

Supplementary Figure 6. Relative expression of all candidates stratified by metastasis status at diagnosis.

Boxplots show the median, first and third quartile for all candidates. Mann-Whitney test results are shown (*p<0.05).

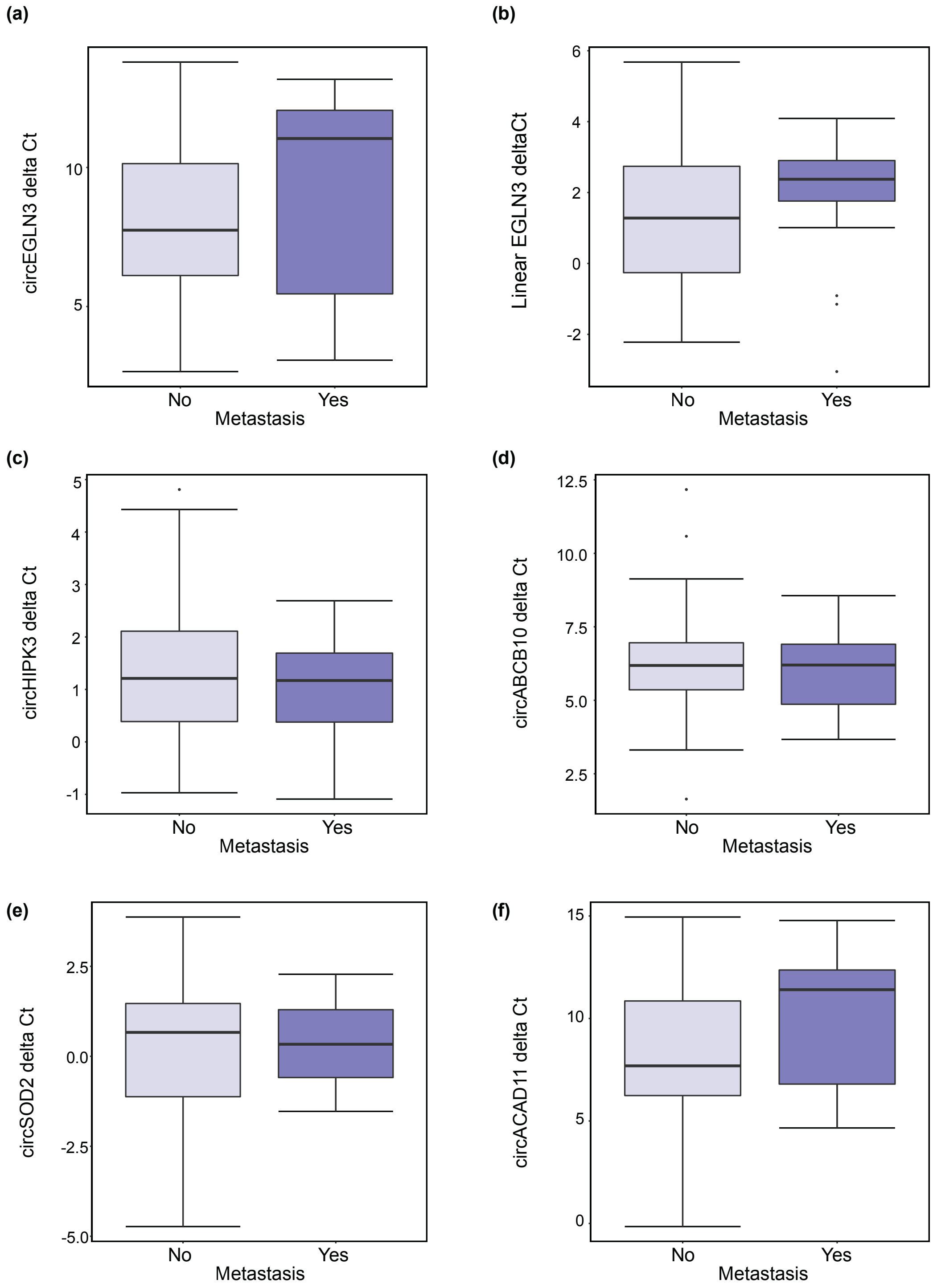

Supplementary Figure 7. Relative expression of all candidates stratified by current metastasis status.

Boxplots show the median, first and third quartile for all RNA candidates. Mann-Whitney analysis was performed to compare all groups.
